# Evaluation of Microorganisms Cultured from Injured and Repressed Tissue Regeneration Sites in Endangered Giant Aquatic Ozark Hellbender Salamanders

**DOI:** 10.1371/journal.pone.0028906

**Published:** 2011-12-19

**Authors:** Cheryl A. Nickerson, C. Mark Ott, Sarah L. Castro, Veronica M. Garcia, Thomas C. Molina, Jeffrey T. Briggler, Amber L. Pitt, Joseph J. Tavano, J. Kelly Byram, Jennifer Barrila, Max A. Nickerson

**Affiliations:** 1 Arizona State University, School of Life Sciences, The Biodesign Institute, Center for Infectious Diseases and Vaccinology, Tempe, Arizona, United States of America; 2 NASA/Johnson Space Center, Habitability and Environmental Factors Division, Houston, Texas, United States of America; 3 Department of Microbiology and Immunology, University of Texas Medical Branch, Galveston, Texas, United States of America; 4 EASI, Wyle Laboratories, Houston, Texas, United States of America; 5 Missouri Department of Conservation, Jefferson City, Missouri, United States of America; 6 University of Florida, Florida Museum of Natural History, Gainesville, Florida, United States of America; Auburn University, United States of America

## Abstract

Investigation into the causes underlying the rapid, global amphibian decline provides critical insight into the effects of changing ecosystems. Hypothesized and confirmed links between amphibian declines, disease, and environmental changes are increasingly represented in published literature. However, there are few long-term amphibian studies that include data on population size, abnormality/injury rates, disease, and habitat variables to adequately assess changes through time. We cultured and identified microorganisms isolated from abnormal/injured and repressed tissue regeneration sites of the endangered Ozark Hellbender, *Cryptobranchus alleganiensis bishopi*, to discover potential causative agents responsible for their significant decline in health and population. This organism and our study site were chosen because the population and habitat of *C. a. bishopi* have been intensively studied from 1969–2009, and the abnormality/injury rate and apparent lack of regeneration were established. Although many bacterial and fungal isolates recovered were common environmental organisms, several opportunistic pathogens were identified in association with only the injured tissues of *C.a. bishopi*. Bacterial isolates included *Aeromonas hydrophila*, a known amphibian pathogen, *Granulicetella adiacens*, *Gordonai terrae*, *Stenotrophomonas maltophilia*, *Aerococcus viridans*, *Streptococcus pneumoniae* and a variety of Pseudomonads, including *Pseudomonas aeruginosa*, *P. stutzeri*, *and P. alcaligenes*. Fungal isolates included species in the genera *Penicillium*, *Acremonium*, *Cladosporium*, *Curvularia*, *Fusarium*, *Streptomycetes*, and the Class Hyphomycetes. Many of the opportunistic pathogens identified are known to form biofilms. Lack of isolation of the same organism from all wounds suggests that the etiological agent responsible for the damage to *C. a. bishopi* may not be a single organism. To our knowledge, this is the first study to profile the external microbial consortia cultured from a Cryptobranchid salamander. The incidence of abnormalities/injury and retarded regeneration in *C. a. bishopi* may have many contributing factors including disease and habitat degradation. Results from this study may provide insight into other amphibian population declines.

## Introduction

The amphibian decline controversy has focused on many factors that affect amphibian populations to varying degrees [1], including habitat loss and degradation, climate change, pollution, increased ultraviolet B (UV-B) radiation, direct exploitation, introduced species and disease, including infectious disease [2], [3]. Although evidence of disease in amphibian populations is not new, early literature on amphibian health in natural populations is quite scattered, deals primarily with anurans and/or local problems, or was initiated because of concerns related to supply and demand and decline of commercial harvest [4]–[6]. In the late 1960's and early 1970's, various biological supply houses noted problems in the commercial supply of amphibians causing Maugh [7] to comment on “the apparent short supply and diseased state of amphibians collected in nature”.

The review of mycoses of amphibians by Reichenback-Klinke and Elkan [8] primarily focuses on *Basidiobolus ranarum* and *Saprolegnia parasitica* and indicates the dearth of knowledge relating to the distribution and importance of microfungi associated with amphibians. With few exceptions, this void continued for the next two decades [9]–[12]. The impetus for research on amphibian microbes became a major focal point of the 1^st^ World Herpetology Congress in Canterbury, England in 1989, where herpetologists shared their observations on declining frog populations and developed initial strategies to investigate the potential problems. The discovery that the chytridiomycete *Batrachochytrium dendrobatidis*, a zoosporic fungus related to infectious oomycete water molds, *Saprolegnia* spp., was capable of causing lethal dermatitis in amphibians led to a proliferation of studies [13]. After reviewing studies of microbes implicated in amphibian population declines, including chytridiomycosis, *Ranavirus* disease, saprolegniosis and *Ribieroia* spp., Daszak et al. [14] concluded that “available data provide the clearest link for the fungal disease amphibian chytridiomycosis”. Herpetological Review dedicated an entire section to amphibian chytridiomycosis geographical distribution [15]–[17].

The advances in research on the secretions, structure, and functions of amphibian integument and their products reveal a remarkable complexity of bioactive secretions and diversity of amines, peptides, alkaloids, bufodienolides, and other compounds [18], [19]. The presence of antimicrobial agents in amphibian skin hypothesized by Csordas and Michl [20] and Croce et al. [21] has led to an increased interest in the relationship of the bacteria and fungi present in the skin of amphibians and the antimicrobial peptides and metabolites that they produce [22]–[30]. Some of these peptides and alkaloids can inhibit the growth of pathogenic fungi [31], [32] and common cutaneous bacteria from the terrestrial salamander *Plethodon cinerus* can inhibit pathogenic fungi [33]. Evidence also suggests that symbiotic bacteria may contribute to innate immune defense of some amphibians [30]. While evidence suggests that these antimicrobial compounds and symbiotic bacteria can provide some level of protection for amphibians against microbial invaders [30]–[33], the connection between disease and amphibian decline has been confirmed for some amphibian populations [34]. However, long-term amphibian studies, especially those including population and environmental data, are so rare that we have very few data to support many claims related to decline or changes in, or the health of, wild amphibian populations [35]. One long-term study subject, the Ozark Hellbender, *Cryptobranchus alleganensis bishopi*, and its habitat within a 4.6 km section of the North Fork of White River (NFWR), has been the subject of numerous investigations since the intensive 110 day surveys conducted during 1969–1971 [36], [37].

In 1969, the 4.6 km research section within the North Fork of White River (NFWR), Ozark County, Missouri, was a crystalline, substantially spring-fed stream located in the least densely human populated area of the second least densely populated county in the state (9 people/sq mi; [38]). Only one rarely used sportsman's cabin graced the banks of the research section in 1969. The springs and occasionally the river were used as drinking water by some locals and visitors. From 1969 to 1980, 169 days of skin-diving surveys, coupled with environmental sampling, were conducted in this section, including some in every year and within every calendar month, but not every month of every year [39]. Population and ecological studies of the aquatic Ozark Hellbender *C. a. bishopi* and its habitat were conducted in this section during 1969–1971 [36], [37]. Other ecological studies included year-round water quality, benthic habitat, macro-invertebrate structure, cottid fish diet studies, and numerous shorter-term studies [36], [40]. These early studies in the NFWR found an immense and healthy population of *C. a. bishopi*, as many as 428 individuals/km, and almost no abnormalities/injuries. Only 2.9% of 479 individuals observed in 1969 were abnormal/injured and they exhibited rapid regeneration capabilities [36], [39], [41]. Additional surveys between 1972 to 1980 continued to show immense and healthy populations of Ozark Hellbenders [27], [42]. All surveys during this time period ranged from between 9–12 individual Ozark hellbenders collected per hour per person (Nickerson et al, unpublished data). Reassessment of the ecological characteristics of the NFWR conducted in 2004–2007 revealed extensive habitat alteration and degradation, including increased land development, siltation, sedimentation, and water quality degradation [43]. Community changes included algal and nuisance aquatic vegetation blooms, otter establishment, and fish and macroinvertebrate community alterations [43]. Canoe use within the NFWR significantly increased [43]. Intensive surveys of the NFWR hellbender population conducted in 2005 yielded only 55 individuals, of which 26 (i.e., 47%) had visible abnormalities/injuries, including loss of limbs, limbs with exposed bones, and degeneration of other tissues which did not regenerate or had remarkably retarded regeneration (J. Briggler, unpublished data). The high prevalence of abnormalities/injuries and the lack of the historically characteristic (rapid) regeneration of injured/affected tissue in hellbenders in the NFWR was the impetus for our examination of the microbial community associated with the observed abnormalities/injuries.

## Results

**Figure 1:**
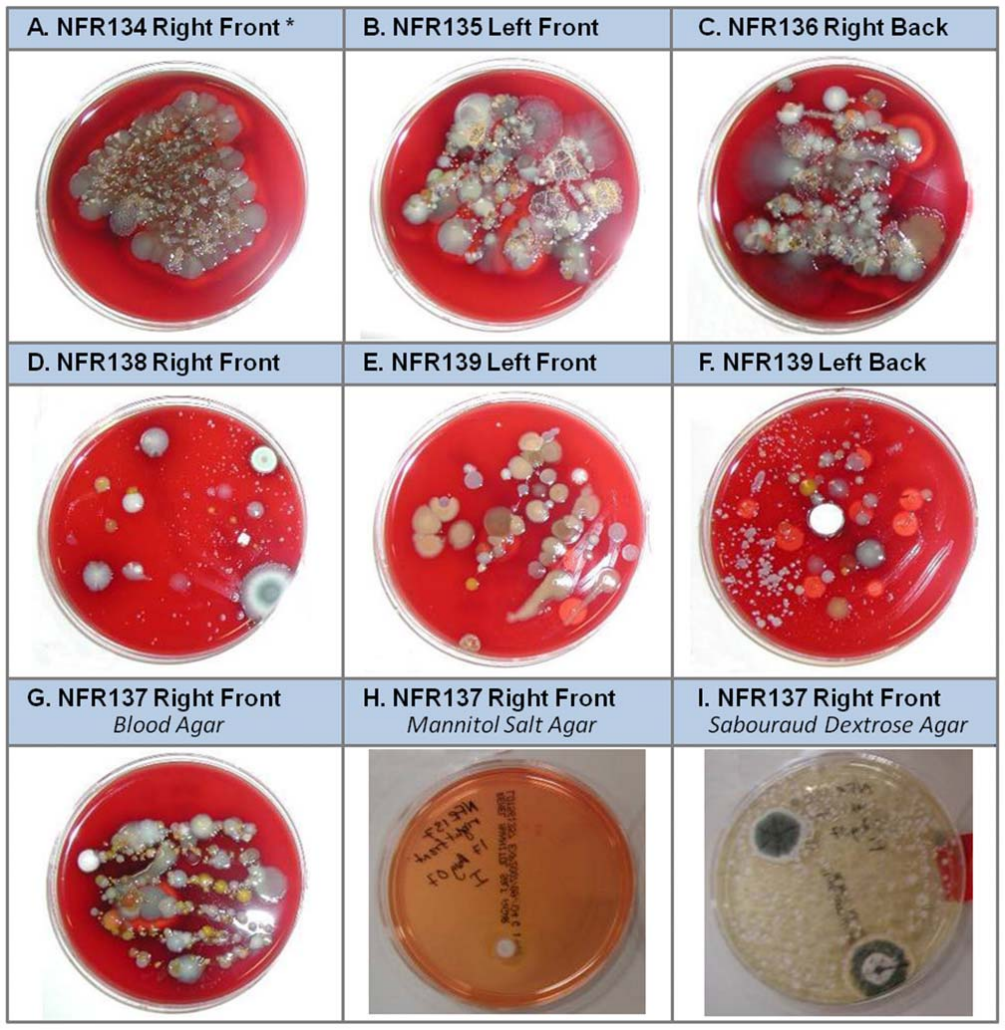
Representative microbial flora cultured from *C. a. bishopi* on three different media. Swabs from injured (or uninjured control) tissues of six adult hellbenders were streaked onto three different microbiological culture media: Sheep's blood agar (A–G), Mannitol Salt Agar (H), and Sabouraud Dextrose Agar (I). * Indicates uninjured control sample.

Our results reflect our strategy to optimize chances for successful culture of potentially pathogenic microorganisms by using three different media for each sample; blood agar (all purpose growth media), mannitol salts (differential and selective media), Sabouraud's (differential and selective media often used to isolate fungi) (Figure 1).

An evaluation of the microbial flora sampled from *C. a. bishopi* indicated the presence of common environmental flora from both abnormal/injured and uninjured limbs. A wide variety of both Gram positive and Gram negative bacteria were isolated. While no consistent pattern of bacterial colonization was observed between uninjured and abnormal/injured body parts (Tables 1 and 2), several interesting microbial associations were observed. The genus *Aeromonas* was identified in 9 separate occasions, although the amphibian pathogenic species *Aeromonas hydrophila*, was identified only once from an abnormal/injured animal (NFWR 139 - lower lip). Likewise, certain organisms that can cause human infection were also identified, including the opportunistic pseudomonal pathogens *Pseudomonas aeruginosa* (NFWR 135 - right back limb), *P. stutzeri* (NFWR 134 - right back; NFWR- 135 right back), *P. alcaligenes* (NFWR – right front), and the pseudomonal-like pathogen, *Stenotrophomonas maltophilia* (NFWR 136 – left front limb). In addition, the poultry pathogen *Riemerella anatipestifer* [44] was also isolated only from abnormal/injured animals. On rare occasion, one bacterial species would represent the vast majority of the colonies from a given abnormal/injured sample as exemplified by the known human pathogen *Granulicatella adiacens* (formerly *Streptococcus adiacens*; [45]), which represented 91% of the 163 bacterial isolates found on the blood agar plate associated with the NFWR 139 lower lip sample. When using Sabouraud Dextrose Agar, only fungi were isolated, except for one plate (from the injured animal NFWR 135 - left front limb) where 4 colonies of *Kocuria kristinae (previously Micrococcus*) were found, which is a common inhabitant on human skin that has been increasingly associated with infectious disease in humans [46].

**Table 1:** Bacteria identified on Blood Agar.

NFWR	Limb sampled	Colony Forming Units	Closest match
134	Right Front*	TNTC	*Aeromonas sobria*
	Right Back	15	*Aeromonas sobria*; *Pseudomonas stutzeri*; *Riemerella anatipestifer*⧫; Unidentified Gram positive species
135	Right Back	TNTC	*Kocuria varians*; *Microbacterium luteolum*⧫; *Pseudomonas stutzeri*; *Pseudomonas aeruginosa*
	Right Front	TNTC	*Roseomonas cervicalis*⧫; *Pseudomonas alcaligenes*; *Brevundimonas diminuta/vesicularis*
	Left Back	TNTC	*Aeromonas sobria* (3 morphologically different colonies)
	Left Front	TNTC	*Kocuria rosea*; *Kocuria kristinae*; *Aeromonas sobria*; *Pseudomonas stutzeri*
136	Left Front	TNTC	*Pseudomonas stutzeri*⧫; *Acinetobacter baumannii*; *Kocuria varians*; *Stenotrophomonas maltophilia* (2 morphologically different colonies)
	Left Back	TNTC	*Aerococcus viridans*; *Aeromonas veronii*⧫;
			*Delftia acidovorans*; *Streptococcus pneumoniae*
	Right Back	TNTC	*Exiguobacterium acetylicum*⧫; *Acenitobacter baumannii*; *Aeromonas sobria; Kocuria varians* (2 morphologically different colonies)
137	Right Front	TNTC	*Kocuria kristinae* (2 morphologically different colonies); *Kocurea rosea*; Unidentified species - unable to isolate (3 morphologically different colonies); Unidentified Gram positive species
	Right Back	TNTC	*Roseomonas cervicalis*⧫; *Kocuria rosea*; *Kocuria varians*; *Myroides* species
	Left Front	TNTC	*Aeromonas sobria*; *Brevundimonas diminuta/vesicularis*; *Kocuria rosea*
138	Left Front	10	*Sphingomonas aurantiaca*; Unidentified species - unable to isolate; Unidentified Gram positive species
	Left Back*	12	*Brevundimonas diminuta/vesicularis*; *Kocuria varians*; *Sphingomonas paucimobilis*
139	Lower Lip	163	*Granulicatella adiacens*; *Aeromonas hydrophila/caviae*; *Bacillus sphaericus/fusiformis*; *Rhizobium radiobacter*
	Left Front	28	*Bacillus lentus*; *Geobacillus thermoglucosidasius/thermodenitrificans*; *Micrococcus luteus/lytae*; *Rhizobium radiobacter*; *Bacillus* species
	Right Front	11	*Sphingomonas paucimobilis*; Unidentified Gram positive species; *Kocuria kristinae*; *Brevundimonas diminuta/vesicularis*; *Dermacoccus*/*Kytococcus* species
	Right Back	19	*Aeromonas sobria*; *Micrococcus luteus*⧫; Unidentified Gram negative species; *Kocuria kristinae*; *Rhizobium radiobacter*; *Ewingella Americana*; *Brachybacterium alementarium*⧫; *Sphingomonas paucimobilis*; *Gordonia terrae*⧫

Colony Forming Units represent the number of microbial colonies counted on each plate. Sample plates which had no growth are not listed. The appearance of different morphologies for singles species is noted. Asterisk (*) indicates control sample from uninjured limb. Diamond (⧫) indicates an isolate identified by 16S sequencing (percent similarity of greater than or equal to 98%). NFWR = North Fork of White River samples.

**Table 2:** Bacteria identified on Mannitol Salt Agar.

NFWR	Limb sampled	Colony Forming Units	Closest match
134	Right Back	4	*Exiguobacterium acetylicum*⧫; *Micrococcus luteus/lylae*
	Right Front*	17	*Kocuria kristinae*; *Micrococcus luteus/lylae*
135	Right Back	11	*Curtobacterium flaccumfaciens*⧫; *Bacillus megaterium*; Unidentified Gram positive species (2 morphologically different colonies)
	Right Front	1	*Micrococcus luteus/lylae*
	Left Back	4	*Staphylococcus hominis*
	Left Front	18	*Brevibacterium casei*⧫; *Kocuria kristinae*; *Staphylococcus sciuri*; *Micrococcus luteus/lylae*
136	Left Front	TNTC	*Bacillus licheniformis*⧫; *Micrococcus luteus/lylae*; *Staphylococcus warneri*
	Left Back	TNTC	*Exiguobacterium acetylicum*⧫; *Bacillus megaterium*; *Staphylococcus hominus/novobisepticus*; Unidentified Gram positive species
137	Right Front	1	*Micrococcus luteus/lylae*
	Right Back	6	*Staphylococcus vitulinus*; *Micrococcus luteus/lylae* (2 morphologically different colonies); Unidentified Gram positive species
	Left Front	TNTC	*Dermacoccus* species/*Kytococcus* species; *Gemella morbillorum*; *Micrococcus luteus/lylae*;
			Unidentified species - unable to isolate
138	Left Front	2	*Dermacoccus/Kytococcus* species
	Left Back*	1	*Bacillus megatarium*
139	Lower Lip	1	*Micrococcus luteus/lytae*
	Left Back	TNTC	*Brevibacterium casei*⧫, *Brevundimonas diminuta/vesicularis* (2 morphologically different colonies); *Brevundimonas diminuta/vesicularis*; *Micrococcus luteus/lylae*
	Left Front	7	*Bacillus lentus*
	Right Front	4	*Pantoea agglomerans*
	Right Back	2	*Pantoea* species

Colony Forming Units represent the number of colonies counted on each plate. Sample plates without growth are not listed. The appearance of different morphologies for singles species is noted. Asterisk (*) indicates control sample from uninjured limb. Diamond (⧫) indicates an isolate identified by 16S sequencing. NFWR = North Fork of White River samples.

Fungal isolates were consistent with common environmental flora from genera that included *Penicillium*, *Streptomycetes*, *Cladosporium*, *Fusarium*, *Acremonium*, *Curvularia*, and the Class Hyphomycetes (Tables 3, 4, and 5).

**Table 3:** Fungi identified on Blood Agar.

NFWR	Limb sampled	Colony Forming Units	Closest match
137	Right Back	60	*Streptomycetes* species
	Left Front	180	*Fusarium* species
138	Left Front	TNTC	*Penicillium* species; *Cladosporium* species; *Streptomycetes* species
	Left Back*	TNTC	*Penicillium* species (2 different species); *Streptomycetes* species
139	Left Back	510	*Hyphomycetes* species; *Penicillium* species;
			*Cladosporium* species (2 different species)
	Right Front	750	*Hyphomycetes* species (2 different species);
			*Cladosporium* (2 different species); *Penicillium* species

Colony Forming Units represent the number of colonies counted on each plate. Sample plates without growth were not listed. Genera with different morphological characteristics suggesting different species are noted. Asterisk (*) indicates control sample from uninjured limb. NFWR = North Fork of White River samples.

**Table 4:** Fungi identified on Mannitol Salt Agar.

NFWR	Limb sampled	Colony Forming Units	Closest match
134	Right Back	90	*Cladisporium* species
			*Penicillium* species
			*Hyphomycetes* species
	Right Front*	30	*Cladosporium* species
135	Right Back	30	*Penicillium* species
	Right Front	30	*Hyphomycetes* species
	Left Back	690	*Cladosporium* species (3 different species)
			*Exophilia* species
			*Hyphomycetes* species
	Left Front	30	*Hyphomycetes* species
136	Left Front	30	*Cladosporium* species
	Left Back	30	*Penicillium* species
	Right Back	60	*Hyphomycetes* species
			*Cladosporium* species
137	Right Front	30	*Penicillium* species
	Right Back	150	*Penicillium* species
	Left Front	180	*Cladosporium* species (2 different species)
			*Hyphomycetes* species (2 different species)
			*Aureobasidium* species
138	Left Back*	60	*Acremonium* species
139	Lower Lip	30	*Cladosporium* species
	Left Back	1100	*Streptomycetes* species
			*Cladosporium* species (2 different species)
	Left Front	210	*Penicillium* species
			*Cladosporium* species
	Right Front	960	*Penicillium* species
			*Cladosporium* species
			*Streptomycetes* species
	Right Back	30	*Penicillium* species

Colony Forming Units represent the number of colonies counted on each plate. Sample plates without growth are not listed. Genera with different morphological characteristics suggesting different species are noted. Asterisk (*) indicates control sample from uninjured limb. NFWR = North Fork of White River samples.

**Table 5:** Fungi identified on Sabouraud Dextrose Agar.

NFWR	Limb sampled	Colony Forming Units	Closest match
134	Right Back	1100	*Penicillium* species (2 different species)
			*Cladosporium* species
	Right Front*	TNTC	*Penicillium* species
			*Hyphomycetes* species
135	Right Back	120	*Penicillium* species
			*Aspergillus* species
			*Hyphomycetes* species (2 different species)
	Right Front	TNTC	*Exophilia* species
	Left Back	600	*Wangiella species*
			*Hyphomycetes* species
			*Penicillium* species
	Left Front	150	*Cladosporium* species (2 different species)
			*Hyphomycetes* species (2 different species)
136	Left Front	60	*Cladosporium* species
			*Streptomycetes* species
	Left Back	30	*Acremonium* species
	Right Back	150	*Fusarium* species
			*Hyphomycetes* species (2 different species)
			*Cladosporium* species
			*Penicillium* species
137	Right Front	TNTC	*Penicillium* species (2 different species)
	Right Back	60	*Curvularia* species
			*Cladosporium* species
	Left Front	TNTC	*Penicillium* species (3 different species)
			*Cladosporium* species
138	Left Front	150	*Penicillium* species
			*Cladosporium* species
			*Hyphomycetes* species
	Left Back*	TNTC	*Penicillium* species
			*Hyphomycetes* species
139	Lower Lip	TNTC	*Penicillium* species
			*Cladosporium* species
	Left Back	330	*Penicillium* species (2 different species)
			*Acremonium* species
			*Sporothrix* species
	Left Front	TNTC	*Cladosporium* species (2 different species)
	Right Front	TNTC	*Cladosporium* species
	Right Back	150	*Cladosporium* species
			*Penicillium* species

Colony Forming Units represent the number of colonies counted on each plate. Sample plates without growth are not listed. Genera with different morphological characteristics suggesting different species are noted. Asterisk (*) indicates control sample from uninjured limb. NFWR = North Fork of White River samples.

## Discussion

The *C. a. bishopi* population decline in the NFWR is well documented and of significant concern [42], [47], [48]. Many reasons for the decline in population and health of the Ozark Hellbender have been suggested, including flooding [39], [49] amphibian harvesting [42], the use of the anesthetic MS-222 (Tricane) [50], [51], the reintroduction and introduction of species including otters and trout [47], [52], habitat alteration and degradation [53], disease including those having a genetic, chemical, or infectious etiology [36], [41], [48], [50], [51], [53]–[61], and the interaction of these factors [47]. Many of these hypothesized causal agents of decline have been investigated to various degrees, yet disease research has largely been limited to *B. dendrobatidis* [59]–[61], and prior to our study, the microbial community associated with the abnormalities typifying the affected hellbenders had not been assessed. As a variety of pathogenic microbes have been linked to amphibian declines, it is critical that the microbial community be examined for all potential disease agents and that research not be initially limited to a single potential infectious agent until causation has been properly evaluated and established.

While our microbiological evaluations in this study indicated common environmental organisms in both abnormal/injured and uninjured Ozark Hellbenders, several opportunistic pathogenic organisms were identified that were associated only with the abnormal tissue/injuries of *C. a. bishopi*, such as *Aeromonas hydrophila*, a known pathogen of amphibians [62], [63], *Granulicatella adiacens* [64], *Stenotrophomonas malophilia* [65], and a variety of opportunistic pathogen Pseudomonad species - *P. aeruginosa* [66], *P. stutzeri* [67] and *P. alcaligenes* [68]. These microbial pathogens are known to form biofilms in the environment and/or in vivo in the infected host. Several of the filamentous fungi isolated in this study, including *Penicillium*, *Fusarium*, *and Cladosporidium* are genera containing opportunistic pathogens that are known to be associated with environmental biofilms [69]–[72]. Multispecies biofilms may interact synergistically yielding an increased resistance to antibacterial agents [73]. While a possible cause and effect role for biofilms in disease progression observed in the Ozark Hellbenders is outside the scope of this study, this observation warrants investigation in future studies. The lack of isolation of the same organism from multiple wounds suggests that none of the organisms identified were the sole etiological agent responsible for the damage to *C. a. bishopi*. If the immune system of the injured *C. a. bishopi* were repressed, it is possible that a combination of the isolated opportunistic pathogens may have contributed to the observed tissue damage. The Gram positive opportunistic pathogen, *Streptococcus pneumoniae*, was isolated from one animal. While the presence of *S. pneumoniae* may be the result of contamination during collection or processing, the genus *Streptococcus* has been found by the EPA downstream of our NFWR research site.

Reintroduced or introduced species may not only be a source of pathogenic microbes in the NFWR, but in some cases may also increase injury, and subsequently infection rates by creating open sores. River otters (*Lutra* or *Lontra canadensis*) used in reintroduction programs are known to carry a suite of pathogenic microbes, including Gram positive *Streptococcus* spp. and Gram negative *Pseudomonas* spp. [74]. Otters reintroduced into Missouri were sourced from Louisiana and could carry many different microbes which are not found in the streams of the Ozark Highlands [75]. We speculate that reintroduced otters may introduce pathogenic microbes into the environment through fecal transmission or direct contact with hellbenders, crayfish (the primary prey of both otters and hellbenders), or other species. In addition, river otters are capable of killing or injuring *C. a. bishopi*. Non-lethal injuries such as bites or scratches yielding open sores may provide a pathway for pathogens. Non-native rainbow trout (*Oncorhynchus mykiss*) and brown trout (*Salmo trutta*) are stocked in the NFWR annually. These trout come from multiple hatcheries with multiple water sources and are transferred between hatcheries, which have known reoccurring water quality issues, including harboring pathogenic microorganisms [76]. While we know of no predation of *C. a. bishopi* by salmonids in the wild, hatchery-raised trout released into the NFWR and other water bodies may serve as a source for pathogens.

Increased recreational use of NFWR may also be a source of hellbender injury and pathogenic microbes. Canoeing and other water activities may disturb or dislodge habitat rocks, inadvertently injuring hellbenders located underneath [43]. Humans may also be a source of pathogens such as *S. pneumoniae*, one of the opportunistic bacteria found in this study.

The rapid regeneration that historically typified injured hellbenders was not apparent in recent studies of the NFWR population. Based on data collected in 1969, hellbender injuries (i.e., tail holes) induced by tagging healed completely with no sign of infection and no visible scars within two months (Nickerson unpublished data). The remarkable regenerative capacity of salamanders has been known since first reported by Spallanzani in 1769 [77]. Regenerative studies have included phylogenetic, seasonal, and environmental analysis of limb regeneration [77]–[79]. Environmental factors that have been considered to affect regeneration include temperature, diet, photoperiod, parasitism, infection, and quality of terrestrial and aquatic microhabitats [79]. Human-induced alterations to the NFWR and surrounding landscape have resulted in changes to the physical-chemical properties of the NFWR, including nutrient-loading, introduction of estrogenic chemical levels, algal blooms, and a microbial content deemed unsafe for full body contact by state and federal agencies [43], [80], [81]. Previous studies investigating the impact of human activities on NFWR water quality revealed relatively high concentrations of total phosphorus (6–52 µg L−^1^) and total nitrogen (0.35–3.06 mg L−^1^) in the 4.6 km research site originally investigated by Nickerson and Mays [80], [81]. The impact of this type of increased nutrient level on the hellbenders was investigated by Solis et al., which focused on a historically populated area, located 11.3 km downstream of the Nickerson and Mays site [80], [82]. Not unexpectedly, the site studied by Solis et al. indicated that nutrient concentrations (including total phosphorous and nitrogen) exceeded the EPA recommended criteria in two thirds of the samples [80]. However, a direct correlation between these elevated levels and abnormalities/disease of the hellbenders was not supported, as all individual concentrations of nutrients and organic chemicals were at much lower levels than any laboratory and field experiments shown to have deleterious effects on amphibians [80]. Likewise, serum samples from *C. a. bishopi* collected at the Solis et al. site were analyzed for possible endocrine disrupting chemicals, however, none were detected at levels above the EPA and Missouri Clean Water Commission criteria for aquatic organisms [82]. Thus, the direct impact of increased chemical levels on the hellbenders remains inconclusive.

The impact of eutrophication associated with human activity was further investigated in a periphyton survey of the NFWR in 2006 to determine if changes in the periphyton communities could be a factor in the Ozark Hellbender decline [81]. Some Periphyton, such as cyanobacteria (i.e., blue-green algae) may cause cutaneous damage, neural and hepatic effects, tumor induction, diarrhea, vomiting, respiratory dysfunction, convulsions and occasionally death [83]. The periphyton community within the NFWR 4.6 km Nickerson and Mays research section consisted of diatoms, chlorophytes, and cyanobacteria [81]. The green algae *Cladophora spp.* achieved relative abundances of >90% of the total periphyton community [81]. Blooms of the benthic, filamentous *Cladophora spp.* are a visible indicator of eutrophication and are linked to phosphorus concentration with 20 µg µL^−1^ being the threshold for *Cladophora* dominance [84], [85]. High nitrate concentrations are an issue with karst topography such as that in the NFWR drainage [81] and increases in human usage and poor sewage facilities. Large *Cladophora* spp. blooms have been a component of the NFWR since at least 1968, but have increased over the decades and large floating algal masses seen during recent summers were not a component of the NFWR 4.6 km section during the early surveys [36]. Increased algal levels are known to increase both biofilm formation and antimicrobial resistance, and *Cladophora spp* mats maintain higher *E. coli* densities than the surrounding aquatic habitat [86], [87]. Green algae (*Cladophora sp.*) mats may have almost ubiquitous populations of *E. coli* and enterococci, which may survive at least six months of drying [88]. A 2007 study of total coliform (TC) bacteria and *Escherichia coli* content was conducted at multiple sites and habitats in the NFWR between Mark Twain National Forest Campground Access and Norfork Reservoir, as well as springs which flow into the NFWR [89]. Total coliform levels exceeded the values deemed safe for full body contact by Missouri Department of Natural Resources (MDNR) in 70 of 94 individual water samples and 25 of the 94 samples also surpassed concentrations of *E. coli* deemed safe for full body contact [89].

Our results do not preclude that an infectious agent caused or exacerbated the tissue damage observed in Ozark Hellbenders, as other microorganisms, which would not grow on the media used in this experiment, may have been present (i.e., the microbial diversity observed in this study is likely a subset of the total microbial diversity). Alternatively, if the immune system of the abnormal/injured *C. a. bishopi* was suppressed, many of the opportunistic pathogens that were isolated in this study, alone or in combination, may have caused infection which was responsible for or served to exacerbate the tissue damage. As such, the increase in incidence of abnormalities/injuries and retardation of tissue regeneration may have multiple contributing factors including changes in the Ozark Hellbenders' susceptibility to infection and exposure to microorganisms. The Ozark hellbender is a federally listed endangered species that has yet to have successfully reproduced in captivity. The unavailability of healthy Ozark hellbenders, small sample size, and conservation status precluded our ability to evaluate all of Koch's Postulates. However, this study provides the most complete analysis of potential microbial stressors on Ozark hellbenders to date and places these findings in the context of habitat alterations. Follow up studies are planned to identify causative mechanism(s) and environmental factors that are contributing to health and population declines in this endangered species.

## Materials and Methods

**Figure 2:**
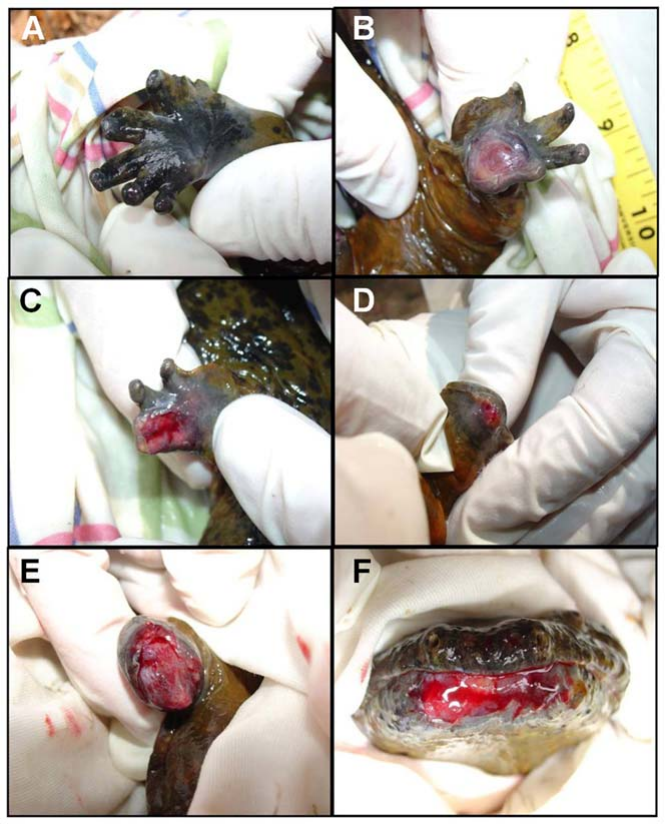
Representative samples of normal and abnormal lesions on Ozark Hellbenders, *Cryptobranchus alleganiensis bishopi*. All individuals sampled were captured from the North Fork of the White River, Ozark County, Missouri on 17 August 2007. A shows a normal left back foot (NFWR 138), B shows lesion on palm of right back foot (NFWR 136), C shows lesion on toes of left front foot (NFWR 136), D shows lesion on right back limb with all toes missing (NFWR 135), E shows lesion on right back limb with all toes missing (NFWR 139), and F shows lesion on lower lip (NFWR 139).

The Ozark Hellbender, *C. a. bishopi*, is now listed as endangered and populations have been extirpated and face extinction in much of the former range. The NFWR currently supports only a very small population of *C. a. bishopi*, of which about 50% within the original NFWR research section of Nickerson and Mays [36], [37] have substantial abnormalities/injuries (J. Briggler unpublished data). On 17 August 2007, we methodically searched a portion of the NFWR by snorkeling and lifting rocks. We located and captured six adult hellbenders, all with abnormalities/injuries (Table 6). Each individual hellbender was placed into a clean bucket filled with river water and then measured, weighed, and individually photographed. All *C. a. bishopi* were visually inspected for the presence of leeches, injuries, or abnormalities. The feet/limbs showing signs of infection (e.g., lesions, sores or exposed bone) were swabbed with sterile, buffered swabs. In addition, the lower lip of one individual with a raw sore was swabbed (Figure 2). Swabs were then streaked onto three different microbiological culture media: sheep's blood agar (SBA), a general all purpose growth medium that supports the culture of a large number of microorganisms and also indicates hemolytic activity; Mannitol Salt Agar (MSA), primarily selective for halo-tolerant bacteria such as staphylococci; and Sabouraud Dextrose Agar (SDX), primarily selective for fungi (Figure 1). Given the very small population of *C. a. bishopi* currently existing in the NFWR and given that no animals without abnormality/injury were captured throughout the duration of this study, two feet showing no signs of infection from two *C. a. bishopi* were swabbed in the same manner and served as uninfected controls. The swabs were streaked onto the different agar plates, and sample plate lids were immediately added and secured with tape. Secured plates were immediately placed into styrofoam coolers with ice packs and transported by vehicle to St. Louis, MO, and flown to the Microbiology Laboratory at the NASA Johnson Space Center (Houston, TX) for microbial identification. Bacterial and fungal isolates were enumerated and then sub-cultured on the medium from the parent culture at room temperature. Bacterial isolates were identified using biochemical analysis with the Vitek 2 system (bioMérieux, Marcy l'Etoile, France). Bacterial isolates that could not be identified by the Vitek 2 system were identified by 16S ribosomal DNA sequencing using a MicroSeq 500 16S rDNA Bacterial Identification Kit (Applied Biosystems, Foster City, CA). Sequences were compared to those on the National Center for Biotechnology Information (NCBI) website for microorganisms. Speciation was reported for isolates having greater than 98% sequence similarity. Fungal isolates were identified by microscopic morphological characteristics [90].

**Table 6:** Ozark Hellbenders, *Cryptobranchus alleganiensis bishopi*, captured and swabbed for microbial flora from the North Fork of the White River, Ozark County, Missouri on 17 August 2007.

Sample No.	Mass (g)	TL (cm)	SVL (cm)	Gender
NFWR 134^1^	559	45.5	31.0	Male
NFWR 135^2^	610	46.0	30.5	Male
NFWR 136^3^	569	45.5	32.5	Female
NFWR 137^4^	690	47.5	47.5	Male
NFWR 138^5^	971	53.0	53.0	Female
NFWR 139^6^	545	48.5	48.5	Male

1 Two sample location (right back limb and right front limb) were swabbed.

2 Four sample locations (all limbs) were swabbed.

3 Three sample locations (right back limb, left back limb, and left front limb) were swabbed.

4 Three sample locations (right back limb, right front limb, and left front limb) were swabbed.

5 Two sample locations (left back limb and left front limb) were swabbed.

6 Five sample locations (all limbs and lower lip) were swabbed.
